# The complete chloroplast genome of *Dendrocalamus liboensis* Hsueh & D. Z. Li 1985 and its phylogenetic analysis

**DOI:** 10.1080/23802359.2024.2306204

**Published:** 2024-01-24

**Authors:** Xue Xu, Mingli Wu, Guangqian Gou, Tanghang Wei, Daoping Yang, Zhaoxia Dai

**Affiliations:** a College of Life Sciences/Institute of Agro-bioengineering, Key Laboratory of Plant Resource Conservation and Germplasm Innovation in Mountainous Region(Ministry of Education), Guizhou University, Guiyang, China; b Forestry Bureau of Wangmo, Wangmo, China; c Forestry Bureau of Tongzi, Tongzi, China; d College of Forestry, Guizhou University, Guiyang, China

**Keywords:** Chloroplast genome, *Dendrocalamus liboensis*, phylogenetic analysis

## Abstract

*Dendrocalamus liboensis* Hsueh & D. Z. Li 1985 is a unique member of the Bambusoideae subfamily found in Guizhou, China. The species has both economic importance and ornamental value. This study represents the first report of the sequencing and assembly of the complete chloroplast genome of *D. liboensis*. The total length of the genome was 139,483 bp, with a conventional quadripartite framework consisting of a large single-copy (LSC) region (83,001 bp in length), a small single-copy (SSC) region (12,896 bp in length), and two inverted repeats (IR) regions (both 21,793 bp in length). Overall, the *D. liboensis* chloroplast genome contained 128 functional genes, including 83 protein-coding genes, 37 tRNAs, and 8 rRNAs. Phylogenetic analysis showed that *D. liboensis* closely resembled *D. sapidus,* with both found on a strongly supported branch of the phylogenetic tree.

## Introduction

First described in 1985, *Dendrocalamus liboensis* Hsueh & D. Z. Li is a member of the *Dendrocalamus* genus that is structurally similar to *D. tsiangii* but with larger rods. *D. liboensis* is only found in Libo County in Guizhou, China, where it is endemic and grows primarily in habitats at altitudes of about 650 m. In common with species such as *Chimonobambusa lactistriata, Indocalamus hirsutissimus,* and *Ampelocalamus scandens*, *D. liboensis* is increasingly threatened by deteriorating natural environmental conditions and a reduction in the size of the wild population (Zhong et al. 2021; Hu et al. 2022; Wu et al. 2022). Bamboo shoots serve as a wild vegetable consumed by local populations and are also used as ornamental garden plants, while bamboo culm is also valued as a building material (Du et al. 2009; Deng and Wang 2006).

*Dendrocalamus* is a member of the subgenus Bambusa within the Poaceae family. Li and Hsueh (1988) classification divides the genus *Dendrocalamus* into two subgenera and five groups, whereas the Forest Code (FOC) divides it into three subgenera and seven groups. Recent systematics studies have shown that *Dendrocalamus* is closely related to *Bambusa* and *Gigantochloa*, which together form the BDG complex (Yang et al. 2008). To date, there has been no analysis of the complete chloroplast genome of *D. liboensis.* Thus, to contribute to the preservation of this species and explore its genetic resources by furthering research efforts, we sequenced, assembled, and analyzed the full *D. liboensis* chloroplast genome. The present report provides a comprehensive description of the *D. liboensis* chloroplast genome and its phylogenetic relationships with other *Dendrocalamus* species.

## Materials and methods

**Figure 1.**
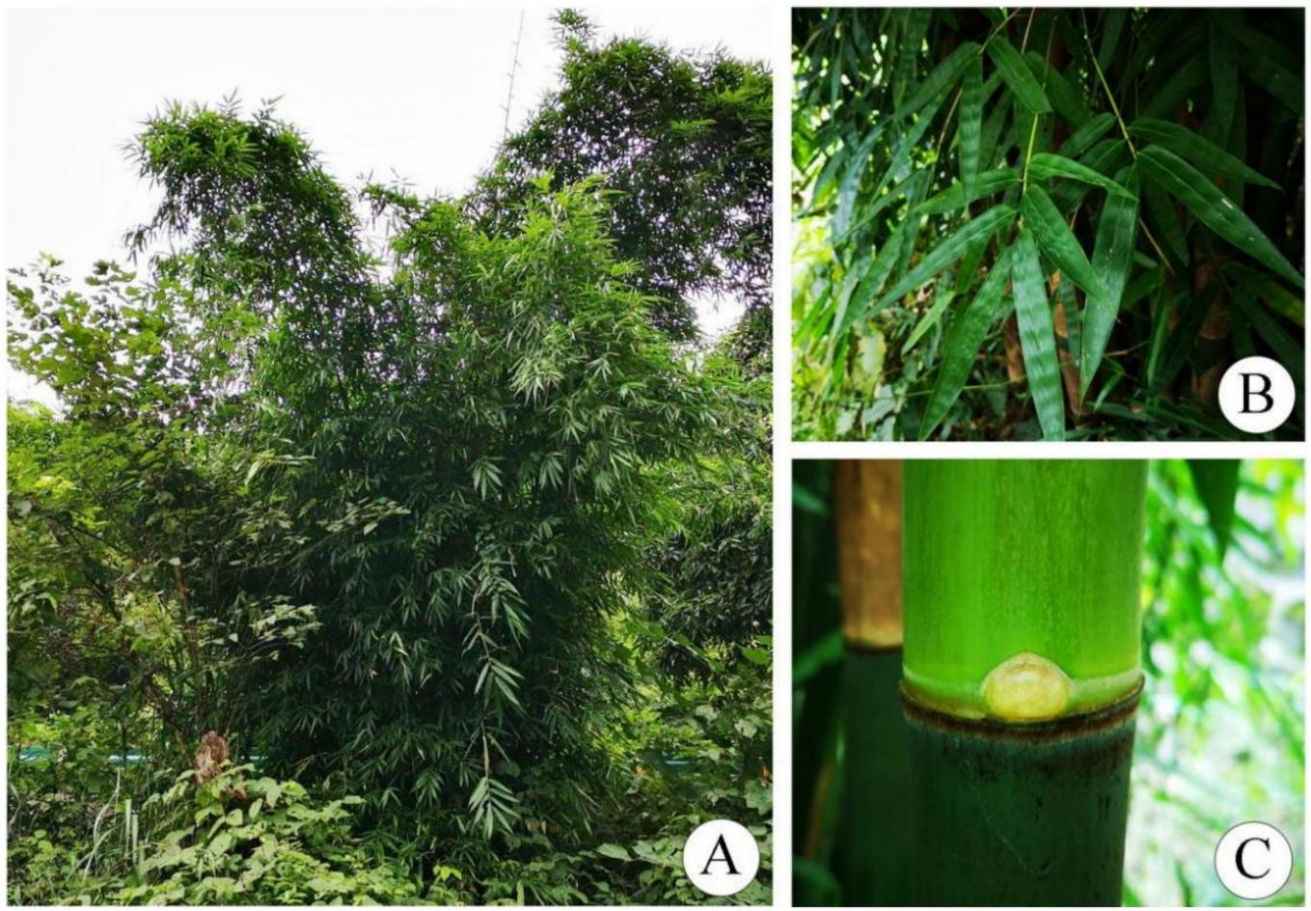
Morphology and habitat map of *Dendrocalamus liboensis* Hsueh & D. Z. Li (These photographs were taken by Guangqian Gou in Libo, Guizhou, China). (A) Clump habit; (B) Leaf branch; (C) Internode. *D. liboensis* is an evergreen bamboo with 8–15 m culms, initially densely white powdery, branches usually from 6th or 7th node up, Central branch dominant and branchlets with 3–9 leaves.

Fresh leaves of *D. liboensis* (Figure 1) were harvested from specimens found in Libo County, Guizhou, China, in April 2022 (25°26′23.24″N, 107°55′0.26″E, altitude: 628 m). The leaves were placed in silica gel and specimens were deposited at the Natural Museum of Guizhou University (contact person: Guang-Qian Gou; email: ggqian106@163.com) under the voucher number GB264. Total DNA was extracted from the leaves using a modified CTAB method (Doyle and Doyle 1987), and the quality and quantity of the DNA was assessed using 1% agarose gel electrophoresis and spectrophotometry (Nanodrop, Thermo Fisher, Waltham, MA, USA), respectively.

The DNA samples were sent to BMK-Beijing (China) for library construction and sequencing on an Illumina HiSeq 2500 instrument (Illumina, San Diego, CA). After quality control, 4.24 Gb of clean data were obtained and assembled into circular contigs with GetOrganelle v.1.7.5 (Jin et al. 2020) and annotated through Plastid Genome Annotator (PGA) (Qu et al. 2019), using *Dendrocalamus brandisii* (MK679786) as the reference (Liu et al. 2020). Genious 9.0.2 was used for the manual correction of the annotated sequencing data (Kearse et al. 2012). The map of the chloroplast genome was drawn using OGDRAW v1.3.1. Mapping raw reads into the chloroplast genome sequence to generated the coverage plot by matplotlib v.3.7.0 (https://matplotlib.org/) (Supplementary Figure 1). The cis-splicing genes and trans-splicing genes were processed using CPGview (Liu et al. 2023). The completed *D. liboensis* chloroplast genome was uploaded to GenBank (accession number OR083041).

Phylogenetic relationships between *D. liboensis* and other *Dendrocalamus* species were explored by downloading 18 complete *Dendrocalamus* chloroplast genome sequences from the NCBI database, as well as the chloroplast genomes of *Bambusa emeiensis* and *Bambusa multiplex* as outgroups. The sequences were aligned using MAFFT (Katoh and Standley 2013), and the optimal model for phylogenetic tree construction was selected with IQtree (Nguyen et al. 2015).

## Results

**Figure 2.**
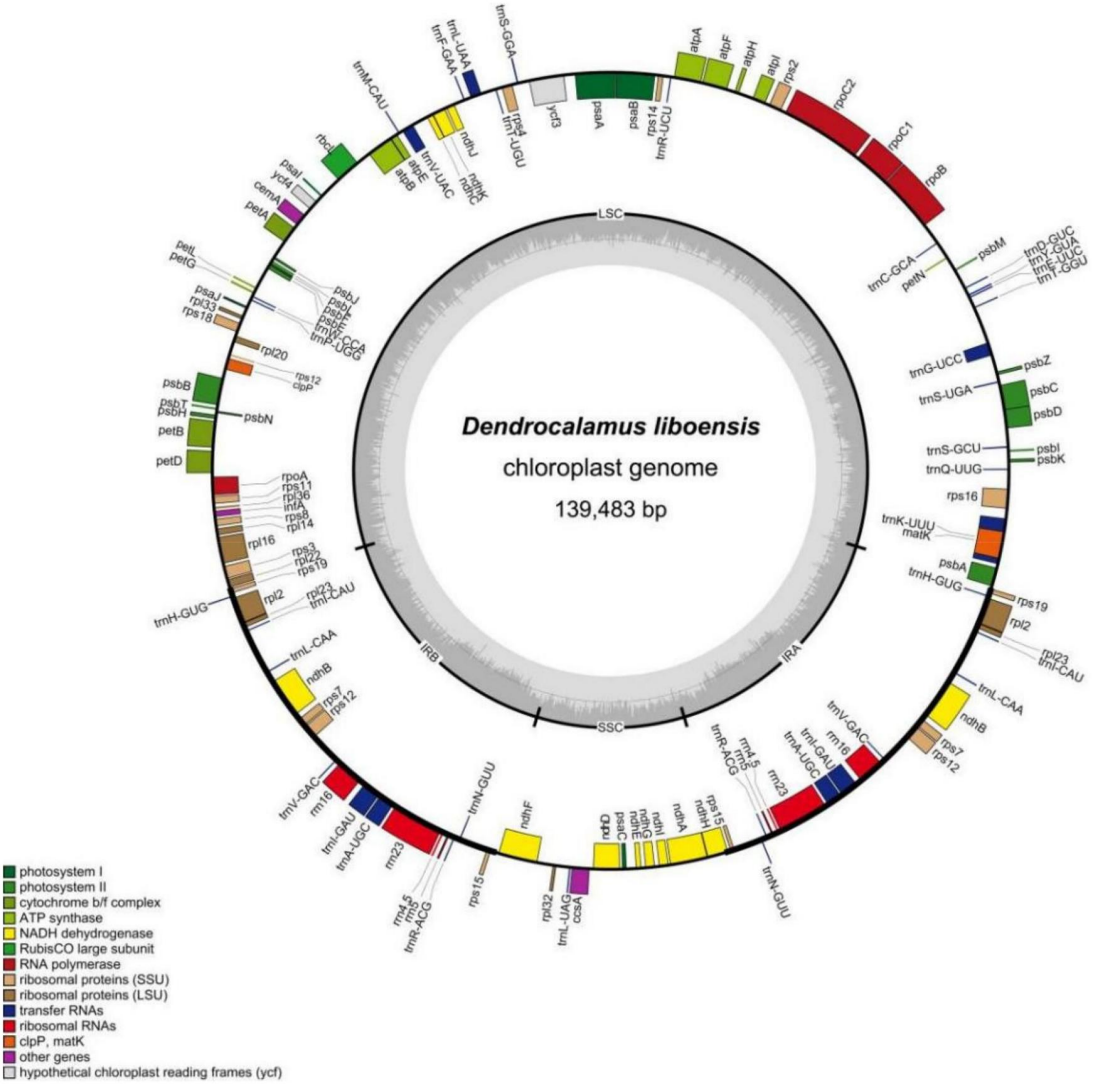
Map of the *D. liboensis* chloroplast genome(GenBank: OR083041). This map was drawn using the OGDRAW. Genes inside the circle are transcribed clockwise, and those on the outside are transcribed counter-clockwise. Genes with related functions are shown in the same color. The darker gray in the inner circle corresponds to DNA G + C content, while the lighter gray corresponds to A + T content. LSC: large single-copy; SSC: small single-copy; IR: inverted repeat.

**Figure 3.**
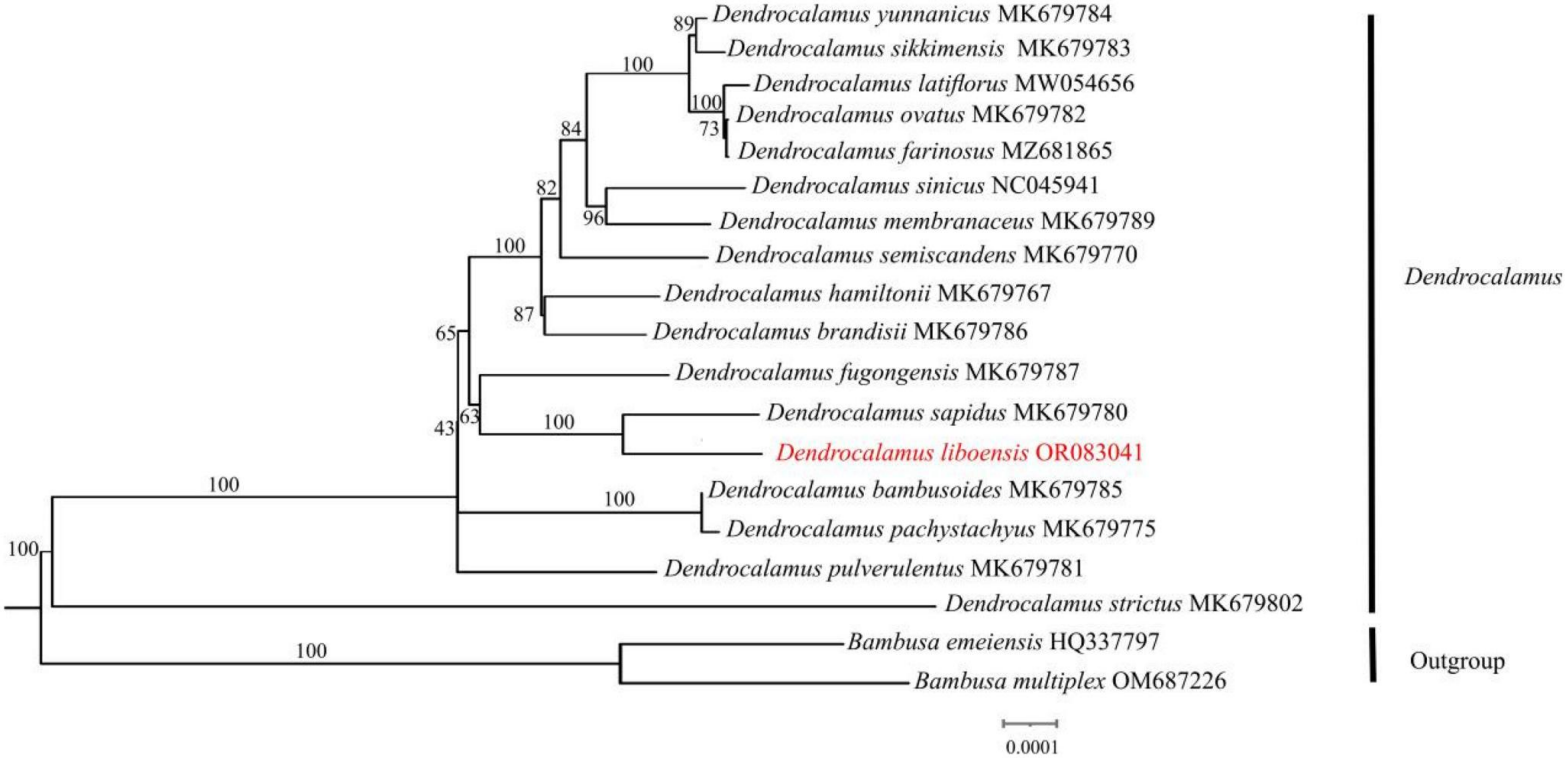
Maximum-likelihood(ML) phylogenetic tree of 19 species by IQtree based on complete chloroplast genomes, with *Bambusa emeiensis* HQ337797 (Zhang et al. 2011) and *Bambusa multiplex* OM687226 as outgroups. The phylogenetic tree was constructed using the maximum-likelihood method (ML) and bootstrap was performed 1000 times. Numbers on the nodes represent the bootstrap support values. The red fonts represents the assembled plastome sequence in this study. The following sequences were used: *Dendrocalamus bambusoides* MK679785 (Liu et al. 2020), *Dendrocalamus birmanicus* MK679772 (Liu et al. 2020), *Dendrocalamus brandisii* MK679786 (Liu et al. 2020), *Dendrocalamus farinosus* MZ681865, *Dendrocalamus hamiltonii* MK679767 (Liu et al. 2020), *Dendrocalamus latiflorus* MW054656(Liu et al. 2020), *Dendrocalamus membranaceus* MK679789 (Liu et al. 2020), *Dendrocalamus fugongensis* MK679787 (Liu et al. 2020), *Dendrocalamus ovatus* MK679782 (Liu et al. 2020), *Dendrocalamus pachystachyus* MK679775 (Liu et al. 2020), *Dendrocalamus pulverulentus* MK679781 (Liu et al. 2020), *Dendrocalamus sapidus* MK679780 (Liu et al. 2020), *Dendrocalamus semiscandens* MK679770 (Liu et al. 2020), *Dendrocalamus sikkimensis* MK679783 (Liu et al. 2020), *Dendrocalamus sinicus* NC045941 (Wang et al. 2019), *Dendrocalamus strictus* MK679802 (Liu et al. 2020), *Dendrocalamus yunnanicus* MK679784 (Liu et al. 2020). Undescribed citations in the legend indicate that the citations have not been published.

The sequencing analysis showed that the *D. liboensis* chloroplast genome had the typical quadrapartite structure observed in most angiosperms. At an overall length of 139,483 bp, with an average coverage depth of 257.63×(Supplementary Figure 1). It showed a typical quadripartite structure, including a large single-copy (LSC) region of 83,001 bp in length, a small single copy (SSC) region of 12,896 bp, as well as two inverse repeats (IR) segments of 21,793 bp each, with GC contents of 37.0%, 33.2%, and 44.2%, respectively (Figure 2). The genome contained 128 functional genes, including 83 protein-coding genes, 37 tRNAs, and 8 rRNAs. Introns were present in 6 tRNA genes (*trn*K-UUU, *trn*I-GAU, *trn*A-UGC, *trn*G-UCC, *trn*V-UAC, and *trn*L-UAA) together with 10 protein-coding genes (*atp*F, *ndh*A, *ndh*B, *pet*B, *pet*D, *rpl*2, *rpl*16, *rps*16, *ycf*3, and *rps*12), with 14 genes containing one intron and two genes (*ycf*3, *rps*12) containing two introns with two copies (Supplementary Figure 2). We constructed an ML tree with 18 complete cp genomes to determine the phylogenetic position of *D. liboensis*. The phylogenetic analysis showed that *D. liboensis* was closest to *D. sapidus,* with both present on a highly supported branch (BS = 100%) (Figure 3).

## Discussion and conclusion

Compared with nuclear and mitochondrial genomes, chloroplast genomes are highly conserved and play an important role in phylogeny and species evolution studies (Du et al. 2017). With the development of high-throughput sequencing technology, chloroplast genome sequences are widely used as super barcodes in species identification and other studies (Li et al. 2021). In this study, the complete chloroplast genome of the economically important and ornamentally valuable species *D. liboensis* was sequenced for the first time and formally submitted to genome repositories. The chloroplast genome structure of *D. liboensis* shows a typical tetrad structure, including the LSC region, SSC region and IR region. The *D. liboensis* chloroplast genome was found to be similar in length and composition to those of other *Dendrocalamus* species (Pei et al. 2022). The geographical distribution of *D. liboensis* and *D. sapidus* is similar, both occurring in southern Guizhou. In addition, these two species have similar morphological characters, including bamboo stems, branches and leaves. The close relationship between the two species is consistent with the results of phylogenetic analyses using chloroplast genome construction. The result enriches the genomic data for the genus *Dendrocalamus*, which will contribute to phylogenetic and evolutionary studies in future.

## Supplementary Material

Supplemental Material

## Data Availability

Genome sequence data that support the findings of this study can be obtained from GenBank of NCBI at https://www.ncbi.nlm.nih.gov/ under accession no. OR083041. The associated BioProject, SRA, and Bio-Sample numbers are PRJNA978368, SRR24793390, and SAMN35387695 respectively.
