# Supplementary material for: The complete chloroplast genome of Dendrocalamus liboensis Hsueh & D. Z. Li 1985 and its phylogenetic analysis
Source: Mitochondrial DNA B Resour. 2024 Jan 24;9(1):158–62. doi: 10.1080/23802359.2024.2306204 (PMC10810639; doi:10.1080/23802359.2024.2306204)
Supplement: Supplemental Material [file TMDN_A_2306204_SM3197.docx]

**Supplemental material**


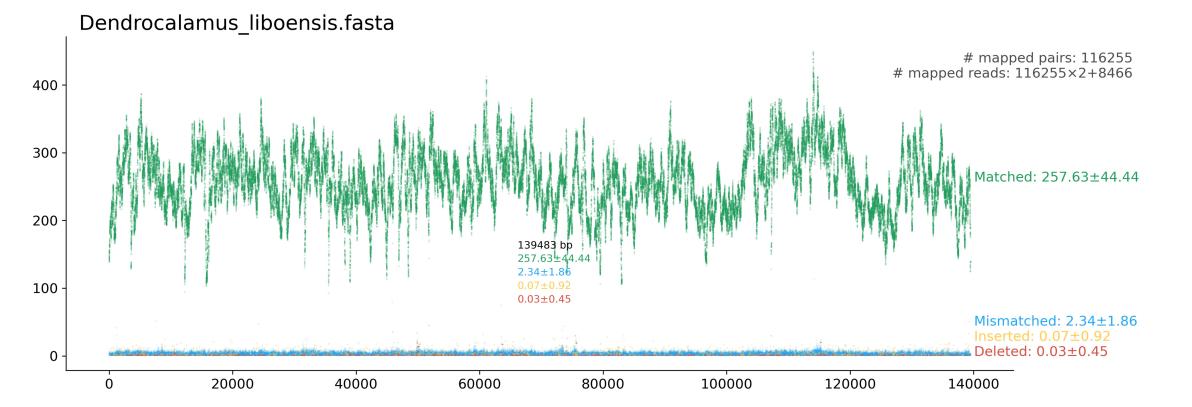


**Supplementary Figure 1.** Coverage plot across the assembled chloroplast genome of *Dendrocalamus liboensis*.


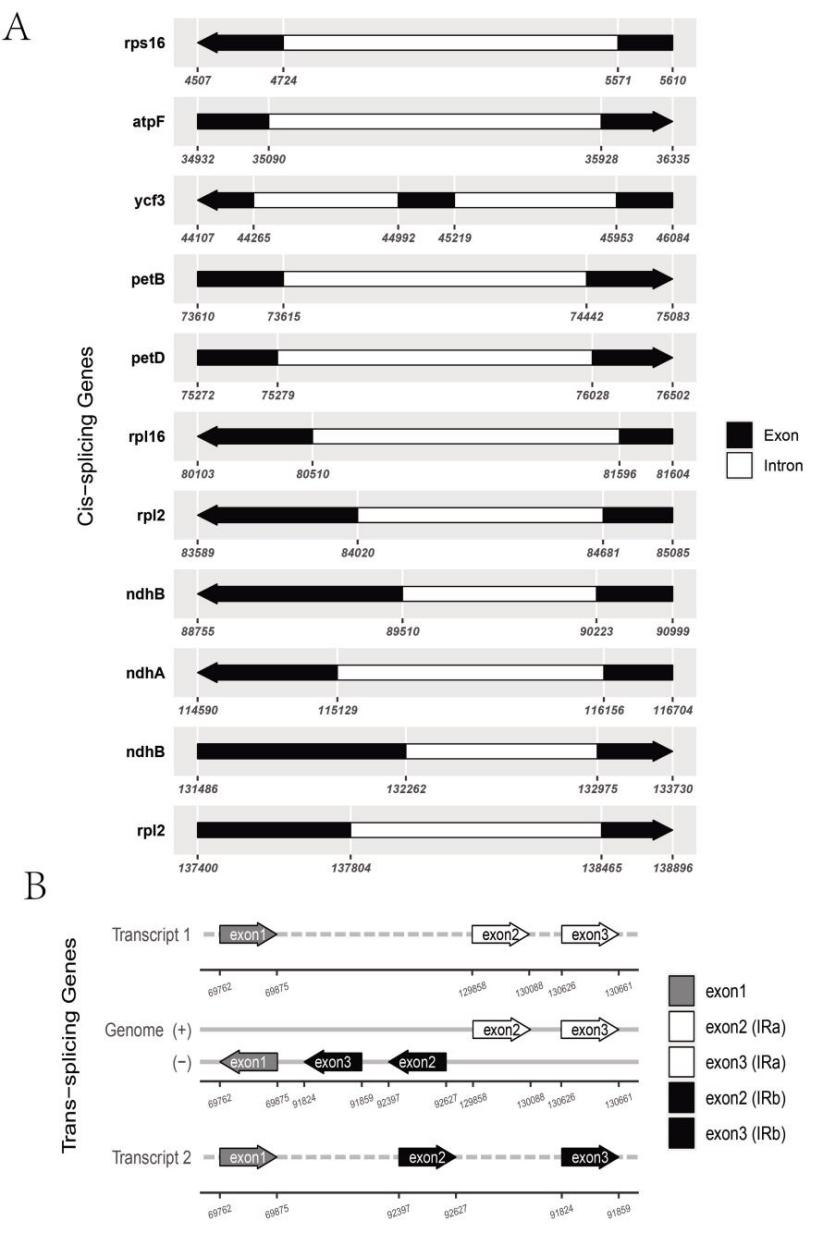


**Supplementary Figure 2.** Schematic map of the cis-splicing and trans-splicing genes. A, cis-splicing genes; B, trans-splicing genes.
